# Nanodelivery of Y-27632 by RGD-modified liposome enhances radioimmunotherapy of hepatocellular carcinoma via tumor microenvironment matrix stiffness reprogramming

**DOI:** 10.7150/thno.114892

**Published:** 2025-07-28

**Authors:** Yang Shen, Zihui Zheng, Xinyao Hu, Zhuolin Zhou, Yangtao Xu, Siyu Wang, Shuhong Yu, Xiaoqin He, Ximing Xu

**Affiliations:** 1 Cancer Center, Renmin Hospital of Wuhan University, Wuhan, Hubei, 430060, China.; 2 Department of Cardiology, Renmin Hospital of Wuhan University, Wuhan, Hubei, 430060, China.

**Keywords:** hepatocellular carcinoma, liposomal platform, radiotherapy, tumor immune microenvironment, matrix stiffness

## Abstract

**Background:** Hepatocellular carcinoma (HCC) causes a significant mortality burden worldwide. Radiotherapy (RT) is the primary locoregional treatment modality for HCC. However, the efficacy of RT in HCC is limited by tumor microenvironment (TME) hypoxia, immunosuppression, and extracellular matrix (ECM) stiffness.

**Methods:** We developed a novel RGD-modified liposomal platform (RGD@LP-Y) that encapsulates the ROCK inhibitor Y-27632 through thin-film hydration. We characterized the RGD@LP-Y by the transmission electron microscope (TEM), UV-Vis spectrophotometer, and dynamic light scattering instrument (DLS). A high-stiffness hydrogel co-culture system mimicking mechanical TME was established to explore the role of RGD@LP-Y on matrix stiffness remodeling. *In vitro* evaluations included cytotoxicity, reactive oxygen species (ROS) generation, mitochondrial function, immunogenic cell death (ICD) markers, and immune cell activation. Mechanistic investigations encompassed matrix stiffness regulation analysis, flow cytometry profiling of pro-inflammatory macrophages, dendritic cell (DC) maturation, transcriptome sequencing, and western blotting. *In vivo* validation used xenograft models treated with intravenous RGD@LP-Y and localized RT. Biosafety was confirmed through organ histology, serum biochemistry analysis, and hemolysis assay.

**Results:** RGD@LP-Y downregulated matrix stiffness markers (YAP/COL1) and activated PI3K/AKT/NF-κB signaling to drive pro-inflammatory macrophage polarization and DC maturation. The synergistic effects were observed in combination with RT. The treatment of RGD@LP-Y and RT inhibited HCC proliferation, induced apoptosis, suppressed mitochondrial respiration, elevated intracellular ROS, and thus enhanced ICD. *In vivo*, RGD@LP-Y+RT demonstrated potent tumor suppression and immune activation without systemic toxicity.

**Conclusion:** RGD@LP-Y enhances RT sensitivity by remodeling ECM stiffness, modulating the hypoxia and immunosuppressive conditions within TME, and enhancing the ICD. The study provides a safe combinatorial approach for HCC therapy.

## Introduction

Primary liver cancer is one of the most common malignant tumors, ranking as the third leading cause of cancer-related mortality worldwide 1, and hepatocellular carcinoma (HCC) is the most common histological type. Despite substantial advancements in local and systemic therapies for HCC, the majority of patients exhibit poor therapeutic responses and ultimately succumb to the disease 2.

As a critical component of local treatment for HCC, radiotherapy (RT) can activate systemic immunity and induce abscopal effects in non-irradiated secondary tumors 3. RT can induce immunogenic cell death (ICD), releasing tumor antigens and damage-associated molecular patterns (DAMPs) such as calreticulin (CRT) and high mobility group box 1 (HMGB1) 4. It also promotes the maturation of dendritic cells (DCs), transforming immunologically "cold" tumors into immunologically "hot" tumors and generating durable antitumor immune responses 5, 6. However, local hypoxia within the tumor microenvironment (TME) and inherent immune suppression can limit the efficacy of RT, resulting in insufficient levels of RT-induced ICD to elicit an adequate anti-tumor immune response 7. Therefore, enhancing the level of ICD is of significant importance for improving the efficacy of RT.

Oxygen plays a significant role in the response to RT 8. Currently, reactive oxygen species (ROS)-based chemo-dynamic therapy can improve oxygenation through Fenton or Fenton-like reactions and may enhance RT efficacy by inducing ICD 9. Multiple strategies can induce ICD by generating ROS, such as using nanozymes 10 or metallic nanomaterials 11, 12 to mimic natural enzymes. Additionally, reducing oxygen consumption by inhibiting cellular respiration can effectively improve tumor oxygenation 13. Nanomaterials can disrupt mitochondrial function, reduce oxygen consumption, and enhance oxygen-dependent RT responses 14.

The highly immunosuppressive tumor microenvironment (TME) is another factor limiting the efficacy of RT. The TME is composed of various stromal cells (epithelial cells, fibroblasts, immune cells, and endothelial cells) and the extracellular matrix (ECM) 15. During tumor progression, the ECM undergoes remodeling and contributes to the stiffening of the tumor 16. Increasing evidence suggests that matrix stiffness in the TME can profoundly impact the tumor immune microenvironment (TIME). For instance, densely arranged ECM can impede the infiltration of immune cells. Matrix stiffness can polarize tumor-associated macrophages (TAMs) into the M2 phenotype, and exacerbate hypoxia in the liver 17-19. Stiff ECM can also trap NK cells and induce PD-L1 through DCs, TGF-β, and cyclooxygenase-2 (COX2). Moreover, a stiff ECM severely restricts cGMP activation, ultimately impairing DC maturation and the activation of effector T cells 17, 20. While studies have attempted to degrade the tumor ECM using drugs or molecules, or combined mechanical therapy with immune checkpoint inhibition to restore TME abnormalities in breast cancer mice 21, 22, these approaches carry risks of systemic toxicity and complications from off-target effects. Novel strategies targeting mechanical reprogramming of the tumor ECM may provide a safer approach to modulate the immunosuppressive TME and potentiate RT outcomes.

The Integrin/RhoA/ROCK axis is a master regulator of ECM biomechanics, orchestrating IFP modulation and solid stress development in tumors 23. The mechanical forces generated by matrix stiffness can activate integrins 24, and the activated integrins can transmit mechanical signals via the Rho signaling pathway. Afterward, the mechanical signals can be transduced into a biological signal, leading to phenotypic changes in HCC cells, such as proliferation and migration, and contributing to therapeutic resistance 23, 25-27. Moreover, matrix stiffness activates the integrin pathway, further remodeling the ECM and forming a vicious cycle that creates a barrier with spatial obstruction and immunosuppressive properties 28. Various strategies focus on targeting ECM abnormal deposition and the pro-tumor signaling 29-31. Therefore, targeting the Integrin/RhoA/ROCK pathway represents a promising strategy to block matrix stiffness-driven signaling, thereby enhancing immune infiltration and creating therapeutic opportunities, critical for amplifying RT-induced ICD.

The Arg-Gly-Asp (RGD) peptide, composed of arginine (R), glycine (G), and aspartic acid (D), exhibits high affinity for integrins (particularly the αvβ3 integrin). Since these integrins are overexpressed in cancer cells and angiogenic regions within the TME, RGD enables tumor tissue targeting 32-34. Additionally, RGD-tagged peptides activate anticancer immune responses by inducing ICD 35. Extensive studies have demonstrated that ROCK inhibitors can suppress tumor cell proliferation, migration, and invasion, induce cell cycle arrest and apoptosis 36, 37, and inhibit mitochondrial respiration 38 in cancer. Furthermore, ROCK inhibitors have been shown to modulate complex components within the TME, including ECM remodeling 39 and immune cell regulation 40. Numerous studies have demonstrated that Y-27632, as a ROCK1 inhibitor, can suppress proliferation, migration, and invasion in multiple tumor types, such as endometrial cancer, oral cancer, and colon cancer 41-43. Thus, our study developed a multifunctional nano platform, RGD@LP-Y, which encapsulates the ROCK inhibitor Y-27632 within RGD-modified liposomes. DSPE-PEG-RGD can target integrins, enhancing tumor targeting and cellular uptake. Additionally, RGD@LP-Y can reprogram the ECM and synergistically block the mechanical signals transmitted through the integrins and Rho/ROCK pathway. It can also activate the PI3K/AKT/NF-κB pathway, inducing pro-inflammatory polarization of macrophages and maturation of DCs. RGD@LP-Y can activate the TIME and effectively sensitize tumors to RT. The combination of RGD@LP-Y with RT can inhibit HCC cell proliferation and migration, induce apoptosis, enhance ROS production, and suppress mitochondrial respiration to improve oxygenation and enhance ICD. This study provides a novel multimodal strategy for the RT of HCC.

## Methods

### Materials

HSPC soybean phosphatidylcholine, DSPE-PEG2000/DSPE-PEG2000-RGD, and cholesterol were provided by Guangzhou Weihua Biotechnology Co., Ltd. (China). Y-27632 was purchased from MedChemExpress Co., Ltd. (USA). HEPES, bis-acrylamide, and ethanolamine were purchased from Sigma-Aldrich Co., Ltd. (USA). Acrylamide (10%), ammonium persulfate (APS), and TEMED were purchased from Serva Co., Ltd. (China). Type I collagen (COL-1) was obtained from Corning Incorporated (USA).

The CCK-8 assay kit and 4′,6-diamidino-2-phenylindole (DAPI) were obtained from Servicebio Co., Ltd. (China). The apoptosis kit was purchased from MultiSciences (Lianke) Biotech Co., Ltd. (China). Calcein-AM/PI kit, tetramethylrhodamine ethyl ester (TMRE) kit, 2′,7′-dichlorofluorescein diacetate (DCFH-DA) kit, and DNA damage detection kit were provided by Beyotime Biotechnology Co., Ltd. (China). The Mouse IL-12 (Interleukin 12) ELISA Kit [E-EL-M3062], Mouse COL1 (Collagen Type I) ELISA Kit[E-EL-M0325], Human TNF-α (Tumor Necrosis Factor Alpha) ELISA Kit [E-EL-H0109], Human IL-6 (Interleukin 6) ELISA Kit [E-EL-H6156], Human IL-1β (Interleukin 1 Beta) ELISA Kit [E-EL-H0149], Mouse IL-6 (Interleukin 6) ELISA Kit [E-EL-M0044], and Mouse TNF-α (Tumor Necrosis Factor Alpha) ELISA Kit [E-EL-M3063] were purchased from Elabscience Biotechnology Co., Ltd. (China).

The following antibodies were obtained from Becton, Dickinson and Company (USA): PerCP-Cy5.5 Rat Anti-Mouse I-A/I-E (M5/114.15.2) [562363], PE-Cy7 Rat Anti-CD11b (M1/70) [552850], APC-Cy7 Rat Anti-Mouse CD45 (30-F11) [557659], and BV421 Rat Anti-Mouse F4/80 (T45-2342) [565411]. The following antibodies were purchased from Elabscience Biotechnology Co., Ltd. (China): FITC Anti-Mouse CD11c Antibody [N418], PE Anti-Mouse CD86 Antibody [GL-1], APC Anti-Mouse CD80 Antibody [16-10A1], APC Anti-Mouse CD3 Antibody [17A2], FITC Anti-Mouse CD4 Antibody [GK1.5], and PE Anti-Mouse CD8a Antibody [53-6.7]. The following antibodies were provided by ABclonal Biotechnology Co., Ltd. (China): PI3 Kinase p85 alpha/beta/gamma mAb [A22996], COX IV Rabbit pAb [A22871], HMGB1 Rabbit pAb [A25444], Calreticulin Rabbit pAb [A1066], CD86 Rabbit pAb [A1199], and YAP1 Rabbit mAb [A19134]. The Recombinant CD4 antibody [ab183685], the Recombinant Anti-CD8 alpha antibody [ab217344], and the Recombinant Anti-F4/80 antibody [ab300421] were purchased from Abcam plc (UK). The Phospho-PI3 Kinase p85 (Tyr458)/p55 (Tyr199) Antibody [4228T] was obtained from Cell Signaling Technology, Inc. (USA). The following antibodies were provided by Proteintech Group, Inc. (China): AKT Monoclonal antibody [60203-2-Ig], Phospho-AKT (Ser473) Monoclonal antibody [66444-1-Ig], NF-κB p65 Recombinant antibody [80979-1-RR], and Phospho-NF-κB p65 (Ser468) Recombinant antibody [82335-1-RR].

The DMEM and RPMI 1640 culture medium and fetal bovine serum (FBS) were obtained from Gibco (USA). Penicillin-streptomycin-amphotericin B antibiotics were purchased from Servicebio Co., Ltd. (China). The HCC cell line Hepa1-6, the human acute monocytic leukemia cell line Tohoku Hospital Pediatrics-1 Cells (THP-1), and the complete culture medium for murine bone marrow DCs (immature DCs) were provided by Procell Life Science & Technology Co., Ltd. (China). The phorbol 12-myristate 13-acetate (PMA) [P6741] was obtained from Beijing Solarbio Science & Technology Co., Ltd. (China). The Active Recombinant Mouse CSF-1/M-CSF Protein [RP01216] was purchased from ABclonal Biotechnology Co., Ltd. (China).

### Preparation of liposomes

Liposomes were prepared using the thin-film hydration method. Briefly, 60 mg HSPC soybean phosphatidylcholine, 20 mg DSPE-PEG2000 or DSPE-PEG2000-RGD, 20 mg cholesterol, and 5 mg Y-27632 were dissolved in 80 ml chloroform. The organic solvent was removed via rotary evaporation (2 h) to form a lipid film. After hydration with 10.3 ml ddH₂O for 30 min, the suspension was ultrasonicated for 5 min and centrifuged at 7200 rpm for 10 min. The liposomes were finally extruded through an Avanti mini extruder to achieve Y-27632-loaded liposomes with a final concentration of 1.6 mg/ml.

### Characterization of liposomes

The liposomes' hydrodynamic size and zeta potential were measured using a dynamic light scattering instrument (DLS, Zetasizer Pro). The morphology of the liposomes was imaged using a JEM-1400Plus transmission electron microscope (TEM). To assess the stability of liposomes, we stored them under four conditions: 4 °C, -20 °C, -80 °C, and in 10% FBS, measuring their hydrodynamic size using DLS every three days.

To determine encapsulation efficiency (EE) and drug loading (DL) capacity, Y-27632's maximum absorption peak was identified at 260 nm using the UV-Vis spectrophotometer (Figure S1A). A standard calibration curve was established using Y-27632 solutions (Figure S1B). Following liposome centrifugation (20,000 g, 40 min), supernatant absorbance at 260 nm was measured for concentration determination.

In vitro release studies of liposomes were conducted over 48 h using the dialysis method. 1 ml each of LP-Y and RGD@LP-Y was introduced into dialysis bags. The dialysis bags were immersed in PBS at pH 7.4 and pH 5.3, respectively, and continuously stirred at 100 r/min and 37 °C. Release medium (1 ml) was collected and replenished with 1 ml of fresh release medium at 0, 1, 2, 3, 4, 6, 8, 12, 24, 36, and 48 h. Drug concentration was measured using a UV-Vis spectrophotometer.

Cells were seeded into six-well plates for the cellular uptake of liposomes. Liposomes were mixed with DiO dye and incubated at 37 °C for 30 min. The cells were washed and centrifuged with PBS at 20,000 g for 40 min. After 24 h, the cells were collected and analyzed using a flow cytometer.

**Cytotoxicity assay.** Hepa1-6 cells were seeded in 96-well plates at a density of 8,000 cells/well, and different concentrations of drugs were added to each well. After 24 h, the medium was replaced with fresh medium, and 10 μl of CCK-8 reagent was added to each well. The plates were incubated at 37 °C in the dark for 1.5 h. The absorbance was measured at 450 nm, and the cell viability was calculated.

**Primary cell isolation.** The femurs and tibias were dissected from male C57BL/6 mice to isolate bone marrow-derived macrophages (BMDMs) and bone marrow-derived dendritic cells (BMDCs). The bone marrow was resuspended in RPMI 1640 complete medium containing 40 ng/ml recombinant CSF-1/M-CSF protein for BMDMs culture and in complete medium for culturing immature DCs in a petri dish for subsequent BMDMs and BMDCs culture.

**Cell culture.** Hepa1-6 cells were cultured in a high-glucose DMEM medium supplemented with 10% FBS and 1% antibiotics. The cells were maintained in a humidified incubator at 37 °C with 5% CO₂. BMDMs and BMDCs were cultured in the complete medium mentioned above. On day 3, the medium was changed by half for both BMDMs and BMDCs. On day 5, the medium was changed by half for BMDMs again, while the loosely adherent cells in the BMDC culture dish were transferred to another dish. Both cell types were cultured for an additional 2 days for subsequent experiments. The THP-1 cells were cultured in RPMI 1640 medium containing 10% FBS and 1% antibiotics and induced into macrophages by adding PMA (20 ng/ml) for 48 h.

**Preparation of gels.** High-stiffness polyacrylamide gels coated with COL-1 were prepared according to the method described by Pelham and Wang 44 with some modifications. First, two glass slides were soaked in 0.1 N sodium hydroxide solution and combined to form a glass chamber. A high-stiffness polyacrylamide gel (16kPa) was prepared by mixing 30% acrylamide and 2% bis-acrylamide in HEPES buffer (pH=8), supplemented with 10% APS and TEMED at a volume ratio of 1/100. A 100 µl aliquot of 0.1 mg/ml COL-1 solution was evenly spread on the surface of the gel and crosslinked at room temperature for 90 min. Subsequently, COL-1 was washed off with PBS, and the gel was blocked in a mixture of 50 mM HEPES buffer and 1% ethanolamine for 30 min. Finally, the gel was incubated overnight at 4 °C in a DMEM medium without FBS for subsequent use. The stiffness of the gel was measured by the Atomic force microscopy (AFM, Bruker, Multimode 8) for the Young's Modulus (YM).

**Coculture assay.** A high-matrix stiffness gel was used to coat the six-well plates, and Hepa1-6 cells were seeded to achieve near 100% confluence. Different materials were then added for treatment for 12 h. Hepa1-6 cells were subsequently cocultured with BMDCs or THP-1 cells after PBS washing.

**Quantitative real-time PCR (qRT-PCR).** Total RNA was extracted using a Trizol reagent, and cDNA was synthesized using a reverse transcription kit. The obtained cDNA was amplified using a 2× Universal Blue SYBR Green qPCR Master Mix. The relative gene expression levels were calculated using the 2^-ΔΔCT^ method. Primer sequences are listed in Table S1.

**Enzyme-linked immunosorbent assay (ELISA):** The cell supernatant was aspirated, and the cells were washed with PBS. Fresh serum-free medium was added, followed by incubation in a 37 °C incubator for several hours. The supernatant was collected, centrifuged to remove debris, diluted to appropriate concentrations, and processed according to the respective ELISA kit instructions. The absorbance was measured at 450 nm, and the concentrations of IL-6, TNF-α, IL-1β, IL-12, and COL1 were calculated according to the standard curve.

**Western blotting (WB).** Cells were washed twice with pre-cooled PBS and lysed using RIPA buffer containing protease inhibitors. The lysates were sonicated and then centrifuged at 12,000 rpm for 10 min at 4 °C to collect the supernatant. Protein concentrations were determined using the BCA assay, and the SDS-PAGE gel was used to separate equal amounts of protein samples. The separated proteins were subsequently transferred onto PVDF membranes and blocked with 5% skim milk for 1-2 h. The membranes were incubated with primary antibodies at 4 °C overnight, followed by incubation with HRP-conjugated secondary antibodies at room temperature for 1 h. Protein bands were visualized using an electrochemiluminescence (ECL) reagent and quantified using ImageJ software.

***In vitro* RT.** Hepa1-6 cells were irradiated using an X-ray irradiator with a local irradiation dose of 6 Gy.

**Measurement of intracellular ROS levels.** Intracellular ROS levels were assessed using the DCFH-DA fluorescent probe. Cells were seeded in 6-well plates and cultured to optimal density. After treatment with 10 μM DCFH-DA at 37 °C in the dark for 30 min, cells were washed three times with PBS to remove residual probes. Fluorescence intensity was captured using a fluorescence microscope and analyzed with ImageJ software.

**Immunofluorescence (IF) staining assay.** Cells were plated in 24-well plates until reaching appropriate confluency. Following two washes with cooled PBS, cells were fixed with 4% paraformaldehyde (30 min), permeabilized with 0.1% Triton X-100 (10 min), and blocked with 10% goat serum (1 h). Primary antibody incubation was conducted overnight at 4°C, followed by incubation with fluorescent secondary antibodies (1 h, room temperature, dark). Nuclei were stained with DAPI (5 min), and fluorescence images were recorded using a fluorescence microscope. Data quantification was performed with ImageJ.

**Mitochondrial membrane potential detection.** Cells in 6-well plates were incubated with 100 nM TMRE working solution at 37 °C in the dark for 30 min. After three washes with pre-warmed PBS, fluorescence signals were visualized under a fluorescence microscope. ImageJ software was used for quantitative analysis.

**Calcein-AM/PI staining.** Cells in 96-well plates were treated with 2 μM Calcein-AM and 4 μM PI (37 °C, 30 min, dark). Following PBS washes, live cells (green fluorescence) and dead cells (red fluorescence) were imaged using fluorescence microscopy. Cell viability was calculated based on the green-to-red fluorescence ratio.

**Apoptosis assay.** Cells in 6-well plates were trypsinized, centrifuged, and resuspended in the binding buffer after PBS washes. Annexin V-FITC (5 μL) and PI (10 μL) were added for 15 min at room temperature in the dark. Apoptotic cell populations were quantified with flow cytometry, and data were analyzed using FlowJo software.

**Wound healing assay.** Confluent cell monolayers in 6-well plates were scratched with a sterile pipette tip to generate linear wounds. After PBS washes, cells were cultured in a serum-free medium. Wound closure was monitored and imaged at 0, 24, 48, and 72 h. Healing rates were calculated using ImageJ by measuring the reduction in wound area.

**Immunogenic cell death (ICD).** ICD markers CRT and HMGB1 were evaluated by IF staining. Cells in 24-well plates underwent fixation, permeabilization, and blocking as described for IF staining. Anti-CRT and anti-HMGB1 primary antibodies were applied overnight at 4 °C, followed by fluorescent secondary antibody incubation (1 h, dark). Fluorescence intensity was captured and analyzed using ImageJ.

**Flow cytometry.** Single-cell suspensions were prepared from mice tumor tissues. The tumor tissues were minced and digested in DMEM containing collagenase I, DNase I, and hyaluronidase at 37 °C for 45 min with shaking. The digested material was then filtered through a 40 µm filter and centrifuged. The cell suspension was gently layered on top of a 70% Percoll working solution at a volume ratio of 2:1 with 30% Percoll and centrifuged at 20 °C for 20 min. The cells from the lower layer were collected for subsequent assays. BMDMs, BMDCs, and THP-1 cells were collected by centrifugation. After trypsinization and washing three times with pre-cooled PBS, the cells were incubated with the corresponding monoclonal antibodies in the dark on ice for 25 min. For the detection of macrophage pro-inflammatory polarization, cells were incubated with monoclonal antibodies against CD45, CD11b, F4/80, and CD86. For the detection of DC maturation, cells were incubated with monoclonal antibodies against CD45, I-A/I-E, CD11c, CD80, and CD86. For Tcell detection, cells were incubated with monoclonal antibodies against CD45, CD3, CD4, and CD8. After washing twice with PBS, cells were resuspended in 200 μl PBS and analyzed using a flow cytometer. Data were processed using FlowJo software to calculate the percentage of positive cells.

**Antitumor efficacy *in vivo*.** The xenograft tumor model was established by subcutaneously injecting tumor cells into C57BL/6 mice. Mice were maintained under sterile conditions, and tumor cells were cultured to the logarithmic growth phase *in vitro*. After washing twice with PBS, cells were resuspended in a serum-free medium, and 1×10^6^ cells were injected into the right flank of each mouse. The animal experiments were approved by the Ethics Committee of Renmin Hospital of Wuhan University (WDRM20241005E).

To evaluate the targeting ability of the materials in vivo, we inject 100 μl of saline solution containing DIO-labeled materials into the tail vein. Under anesthesia, the immunofluorescence was detected in the tumor at various time points using the IVIS imaging system. At 24 h post-injection, the mice were euthanized, and the fluorescence imaging for major organs and tumor tissues was collected to examine drug distribution in vivo.

When the tumor volume approached 100 mm³, different materials were administered via tail vein injection either alone or in combination with RT, and the mice were randomly assigned to eight groups: PBS, PBS+RT, Y-27632, Y-27632+RT, LP-Y, LP-Y+RT, RGD@LP-Y, and RGD@LP-Y+RT. During the observation period, tumor size and body weight of the mice were monitored every other day. Tumor volume was measured using the formula V= (L×W^2^)/2 (L, length of the tumor; W, width of the tumor). At the end of the experiment, the mice were euthanized, and tumor tissues were collected for further analysis. Survival time was recorded as the duration from tumor inoculation until euthanasia when the tumor volume reached 1500 mm³ or until death.

**Biosafety analysis.** Healthy C57BL/6 mice were used, and 100 µl of different materials were injected into the tail vein. On day 14, blood was collected from the retro-orbital venous plexus to assess biochemical functions, and major organs (heart, liver, spleen, lung, and kidney) were harvested for hematoxylin and eosin (H&E) staining. We then collect blood from healthy mice, centrifuge it, take the bottom blood cells, add 1 ml of materials or PBS/ddH₂O, and observe the hemolysis.

**Statistics analysis.** All statistical analyses were performed using GraphPad Prism software (version 8.0). Experimental data are presented as the mean±standard deviation (SD). Statistical differences between the two groups were evaluated using Student's t-tests, and ordinary one-way ANOVA with a Tukey's test (or with a Dunnett's test) was used for multiple group comparisons. The survival of the mice was compared using the Kaplan-Meier method, and the differences between survival curves were tested using the log-rank test. * P < 0.05, ** P < 0.01, *** P < 0.001, ns, not significant.

## Results

### Construction and characterization of RGD@LP-Y

**Figure 1:**
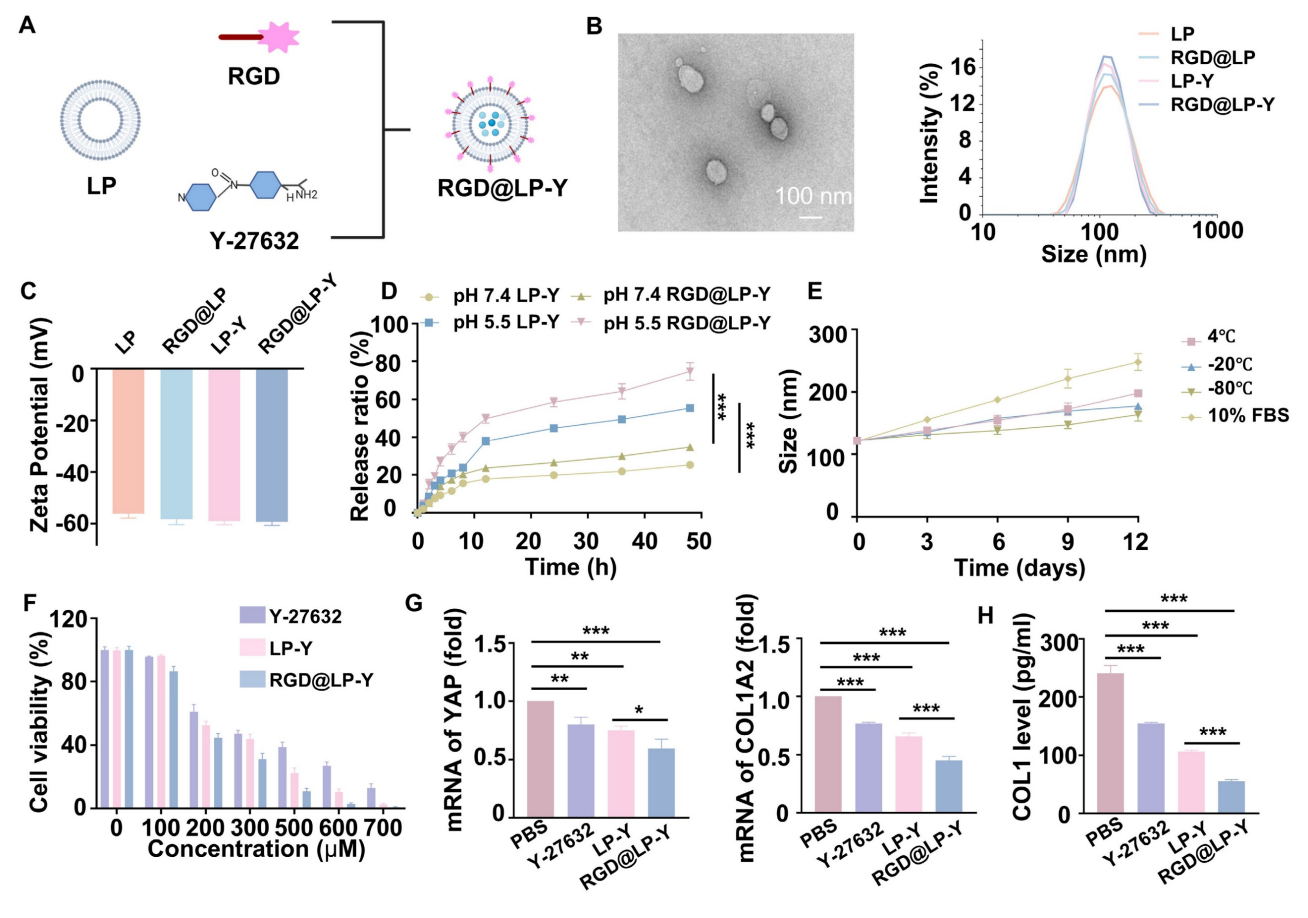
** Preparation and characterization of RGD@LP-Y.** (**A**) Schematic illustration of RGD@LP-Y preparation. (**B**) TEM observation of RGD@LP-Y morphology. (**C**) Zeta potential of RGD@LP-Y. (**D**) The cumulative release of LP-Y and RGD@LP-Y at different pH values (pH 7.4 and 5.5). (**E**) Variations in the particle size of RGD@LP-Y under conditions of 4 ℃, -20 ℃, -80 ℃, and 10% serum after 12 days. (**F**) Relative viability of Hepa1-6 cells treated with different liposomes measured by CCK-8 assay. (**G**) mRNA expression levels of YAP and COL1A2. (**F**) Detection of COL1 level in Hepa1-6 cell supernatant by ELISA. LP, liposomes without RGD modification; RGD@LP, RGD-modified liposomes; LP-Y, liposomes encapsulating Y-27632 but without RGD modification; RGD@LP-Y, RGD-modified liposomes encapsulated with Y-27632. Values represent mean±SD. *P < 0.05, **P < 0.01, ***P < 0.001. ns: not significant.

The construction of RGD@LP-Y is shown in Figure 1A. The thin layer dispersion method was used, involving the preparation of materials such as soybean phospholipids, an RGD-modified phospholipid (RGD-DSPE-PEG2000), cholesterol, and Y-27632 to form the RGD-modified liposomes encapsulated with Y-27632 (RGD@LP-Y). Liposomes without RGD modification (LP), RGD-modified liposomes (RGD@LP), and liposomes encapsulating Y-27632 but without RGD modification (LP-Y) were also constructed for further analysis. The RGD@LP-Y's DL capacity was 53.14%, with an EE rate of 93.04%. Examination of the size, dispersion, and shape of RGD@LP-Y using TEM revealed that it had a uniformly dispersed elliptical spherical size, and particle size measurements showed that the particle sizes of LP, RGD@LP, LP-Y, and RGD@LP-Y were all around 100 nm (Figure 1B). Zeta potential measurements indicated that the liposomes exhibited potentials around -60 mV (Figure 1C). The RGD peptide modification did not significantly change the liposome particle size and Zeta potential. At pH 7.4, LP-Y and RGD@LP-Y released 25.30% and 34.7% of Y-27632, respectively. In contrast, at pH 5.5, LP-Y and RGD@LP-Y released 55.37% and 74.67% of Y-27632, respectively (Figure 1D). This indicates that both LP-Y and RGD@LP-Y possess favorable pH-responsive release characteristics. The particle size of RGD@LP-Y did not change substantially under conditions of 4 °C, -20 °C, -80 °C, and in 10% FBS (Figure 1E), demonstrating the favorable stability of RGD@LP-Y.

### Pharmacotoxicity and uptake of liposomes *in vitro*

The pharmacotoxicity of liposomes was further evaluated for safety by assessing their effect on the viability of Hepa1-6 cells (Figure 1F). We measured cell viability at RGD@LP-Y concentrations ranging from 0 to 700 μM, and it was ultimately determined that Y-27632 encapsulated in liposomes of 100 μM could meet the safety requirements. By staining the liposomes with DIO fluorescent dye, we observed that RGD@LP-Y possessed better cellular uptake than the LP-Y and PBS groups (Figure S1C). This demonstrated that RGD-modified liposomes have good targeting ability.

### RGD@LP-Y inhibits matrix stiffness to activate the immunogenicity

To verify the effect of RGD@LP-Y on matrix stiffness, we prepared gels coated with COL1 at a stiffness of 16 kPa, as previously described in the methods section, to serve as a high matrix stiffness background. We measured the stiffness of the gels using the AFM to get the YM before and after the addition of RGD@LP-Y, and found that the stiffness of the gels decreased from 16.043±0.772 kPa to 9.981±0.588 kPa (Table S2), demonstrating that RGD@LP-Y can significantly reduce matrix stiffness. We then seeded the HCC cell line Hepa1-6 onto the gels. The expression of Yes-associated protein (YAP) and Collagen Type I Alpha 2 Chain (COL1A2), proteins that correlated with cellular mechanics, were reduced in cells treated with RGD@LP-Y (Figure 1G), and the level of COL1 secreted by cells was also decreased (Figure 1H), which suggests that the matrix stiffness of the tumor cells was reduced. The effect was better than that of LP-Y, which may be related to the inhibition of related integrins by the RGD peptide and the increase in the cellular uptake of liposomes 45.

**Figure 2:**
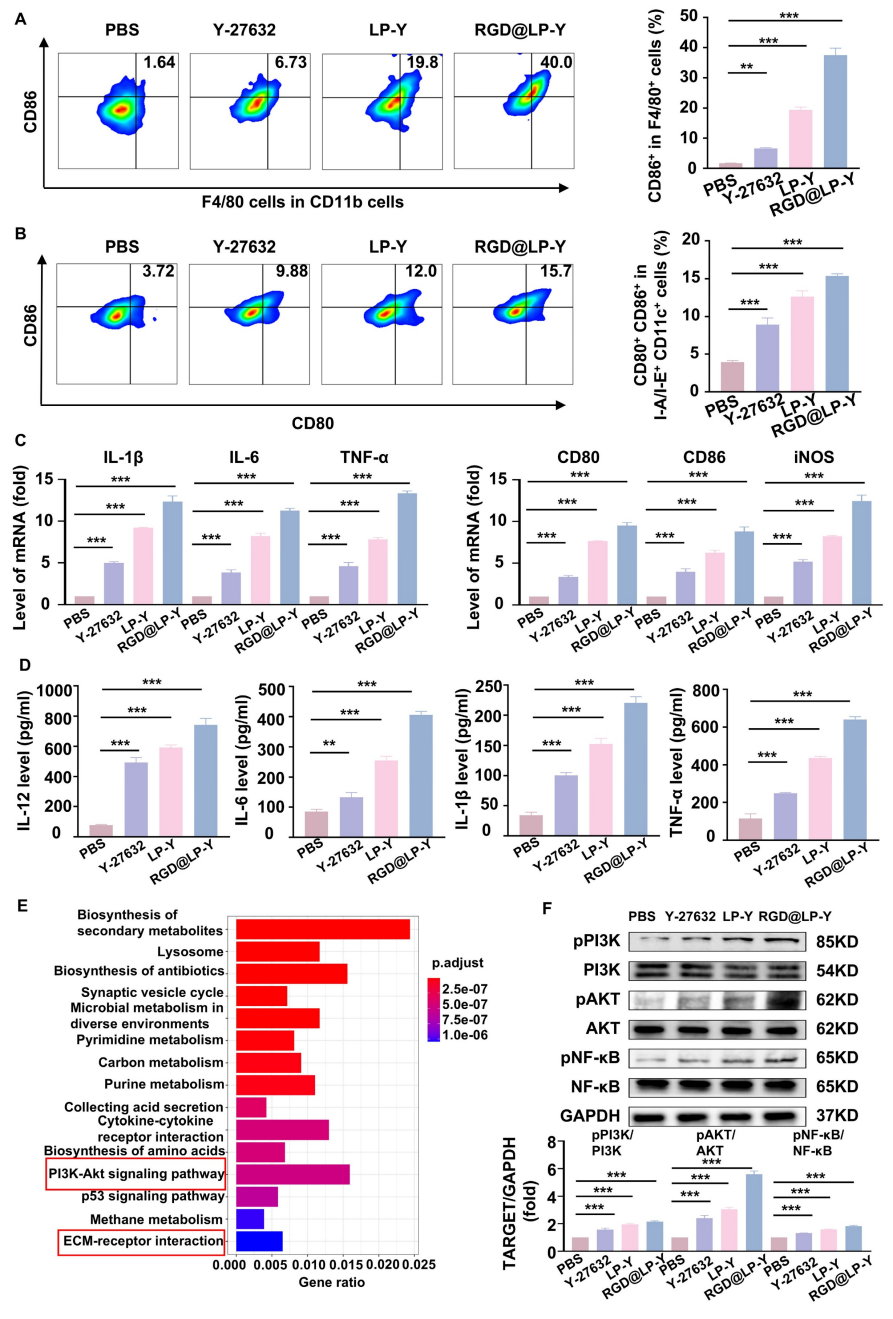
** RGD@LP-Y alleviates immune suppression by reprogramming matrix stiffness.** (**A-B**) Representative flow cytometry plots and quantitative analysis of pro-inflammatory macrophages (CD86^+^) and mature DCs (CD80^+^/CD86^+^) in THP-1 cells and BMDCs co-cultured with tumor cells treated with high-stiffness gel. (**C**) mRNA expression levels of IL-1β, IL-6, TNF-α, CD80, CD86, and iNOS. (**D**) Detection of IL-12 level in BMDC, and IL-6, IL-1β, and TNF-α levels in THP-1 cell supernatant by ELISA. (**E**) KEGG enrichment analysis of differentially expressed genes in Hepa1-6 cells treated with RGD@LP-Y. (**F**) Representative WB bands and quantitative analysis of pPI3K, PI3K, pAKT, AKT, pNF-κB, and NF-κB in Hepa1-6 cells treated with different liposomes. LP, liposomes without RGD modification; RGD@LP, RGD-modified liposomes; LP-Y, liposomes encapsulating Y-27632 but without RGD modification; RGD@LP-Y, RGD-modified liposomes encapsulated with Y-27632. Values represent mean±SD. *P < 0.05, **P < 0.01, ***P < 0.001. ns: not significant.

Previous studies have shown that increased matrix stiffness suppresses immunogenicity, prevents DC maturation and effector T cell activation, and promotes the conversion of TAMs to a M2 phenotype 20. To further investigate the effects of Y-27632 on immunogenicity under conditions of high matrix stiffness, we co-cultured THP-1 cells and BMDCs with Hepa1-6 cells at a ratio of 1:10 2. To minimize the impact of the matrix on floating immune cells, THP-1 cells and BMDCs were added only after the gel surface was completely covered by the HCC cell line, thereby avoiding direct contact between these cells and the gel substrate. We found that RGD@LP-Y was able to reverse the inhibitory modulation of the TIME by high matrix stiffness, promote the transformation of TAMs to the pro-inflammatory phenotype (Figure 2A), and encourage the maturation of DCs. (Figure 2B). The expression of pro-inflammatory macrophage biomarkers (IL-1β, IL-6, TNF-α, CD80, CD86, iNOS) was increased. Furthermore, levels of IL-12 secreted by DCs, along with increased secretion of IL-6, IL-1β, and TNF-α by macrophages, were also elevated (Figure 2C, D), confirming the maturation of DCs and the pro-inflammatory polarization of macrophages.

To further explore the mechanisms involved, we collected Hepa1-6 cells treated with RGD@LP-Y and PBS for transcriptome sequencing and found that the differential genes between the two groups of cells were mainly enriched in the PI3K/AKT pathway and the ECM receptor-related pathway (Figure 2E). PI3K/AKT is a downstream pathway of ROCK, and a previous study has demonstrated that the inhibition of ROCK could activate the PI3K/AKT pathway 46. Subsequently, we verified by WB experiments that RGD@LP-Y could activate the PI3K/AKT/NF-κB pathway (Figure 2F). RGD@LP-Y may exert the immune-activating effects by blocking signals transmitted through the Rho/ROCK due to matrix stiffness and activating the PI3K/AKT/NF-κB signaling pathway. The activation of the PI3K/AKT/NF-κB pathway promotes the pro-inflammatory polarization of macrophages and the maturation of DCs, thus alleviating the immune-suppressive TME mediated by matrix stiffness.

### RGD@LP-Y-mediated *in vitro* antitumor effects and sensitization to radiotherapy

**Figure 3:**
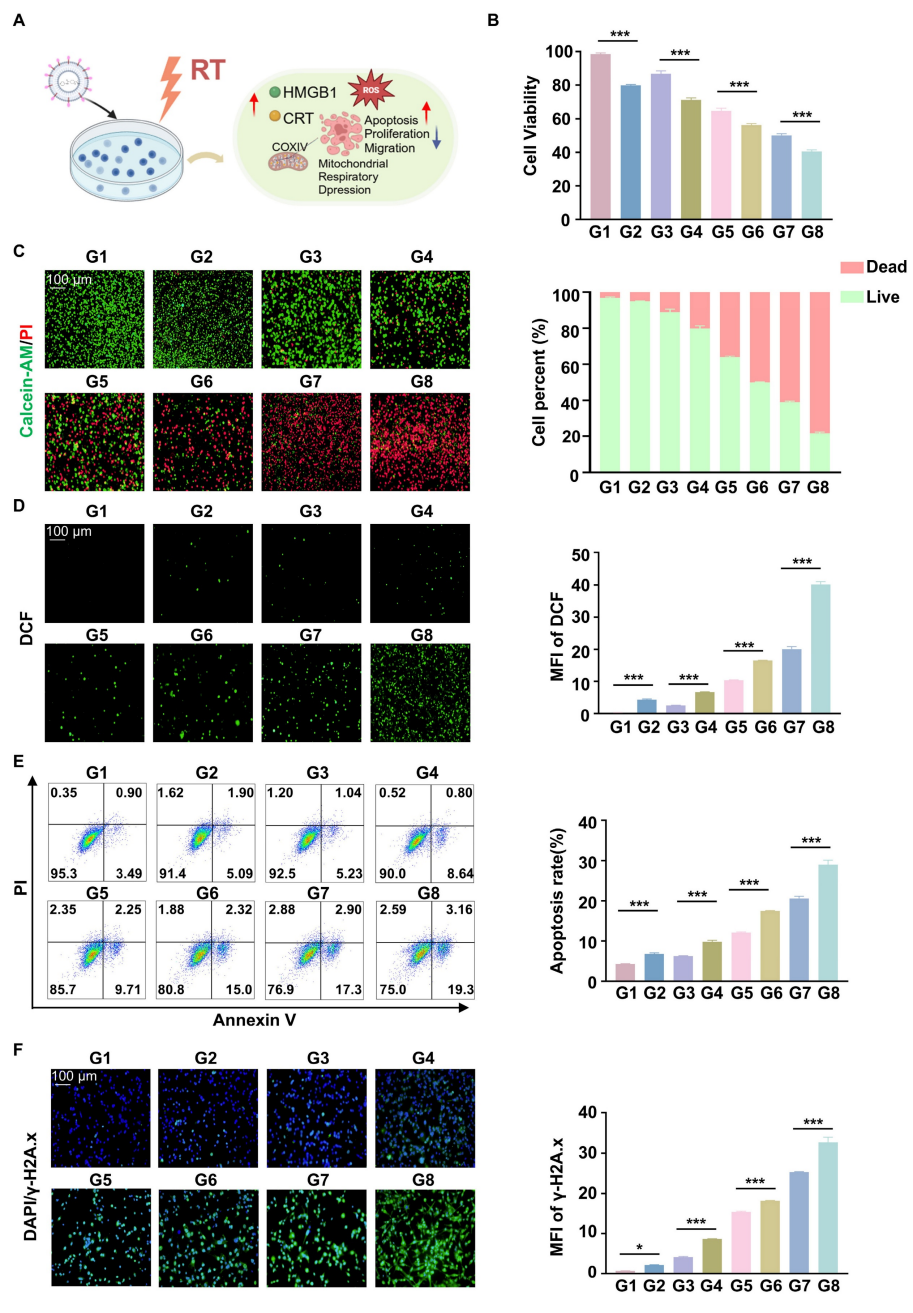
**Anti-tumor and RT sensitizing effects of RGD@LP-Y *in vitro***. (**A**) Schematic illustration of the study of the mechanism of RGD@LP-Y sensitizing RT *in vitro*. (**B**) Relative viability of cells treated with different materials and X-ray irradiation, measured by CCK-8 assay. (**C**) Representative images and quantitative analysis of the survival rate of cells treated with different liposomes and X-ray irradiation, determined by live/dead cell staining. (**D**) Representative immunofluorescence images and quantitative analysis of ROS levels in cells treated with different materials and X-ray irradiation, detected using a DCFH-DA probe. (**E**) Representative flow cytometry plots and quantitative analysis of apoptosis in cells treated with different liposomes and X-ray irradiation, assessed by Annexin V-FITC/PI staining kit. (**F**) Expression of DNA damage marker γ-H2A.x and mean fluorescence intensity (MFI) quantitative analysis in cells treated with different materials and X-ray irradiation. Values represent mean±SD. *P < 0.05, **P < 0.01, ***P < 0.001. ns: not significant. G1, PBS; G2, PBS+RT; G3, Y-27632; G4, Y-27632+RT; G5, LP-Y; G6, LP-Y+RT; G7, RGD@LP-Y; G8, RGD@LP-Y+RT.

The cells were treated with CCK8 assays showed that the cell viability of Hepa1-6 was inhibited when cells were treated with RGD@LP-Y (100 μM), and the lowest cell viability was observed when RGD@LP-Y and RT were combined (Figure 3B). This suggests that RGD@LP-Y can inhibit cell proliferation and that cell proliferation inhibition is strongest when combined with RT. Live-dead staining experiments also confirmed this. It showed that the percentage of dead cells gradually increased in the PBS, Y-27632, LP-Y, and RGD@LP-Y groups and reached the highest dead/live cell ratio in the RGD@LP-Y+RT group (Figure 3C), suggesting a gradual increase in the level of cell killing. The wound-healing assay also demonstrated that RGD@LP-Y could inhibit the migration of HCC cells, and the strongest inhibition of migration was achieved after combination with RT (Figure S2). The anti-tumor effect of RT is mainly dependent on the activation of the generation of large amounts of ROS. By detecting cellular ROS levels, it was found that RGD@LP-Y can generate high levels of ROS and was able to increase ROS levels most significantly when combined with RT (Figure 3D). Moreover, RT can also induce apoptosis, thereby suppressing tumor cells. RGD@LP-Y can promote HCC cell apoptosis, and the promotion level was the strongest in the RGD@LP-Y+RT group (Figure 3E). This suggests that RGD@LP-Y and RT in combination can induce more apoptosis. The level of DNA damage was also the most significant in RGD@LP-Y in combination with the RT group (Figure 3F).

### RGD@LP-Y acts on cellular oxygenation

**Figure 4:**
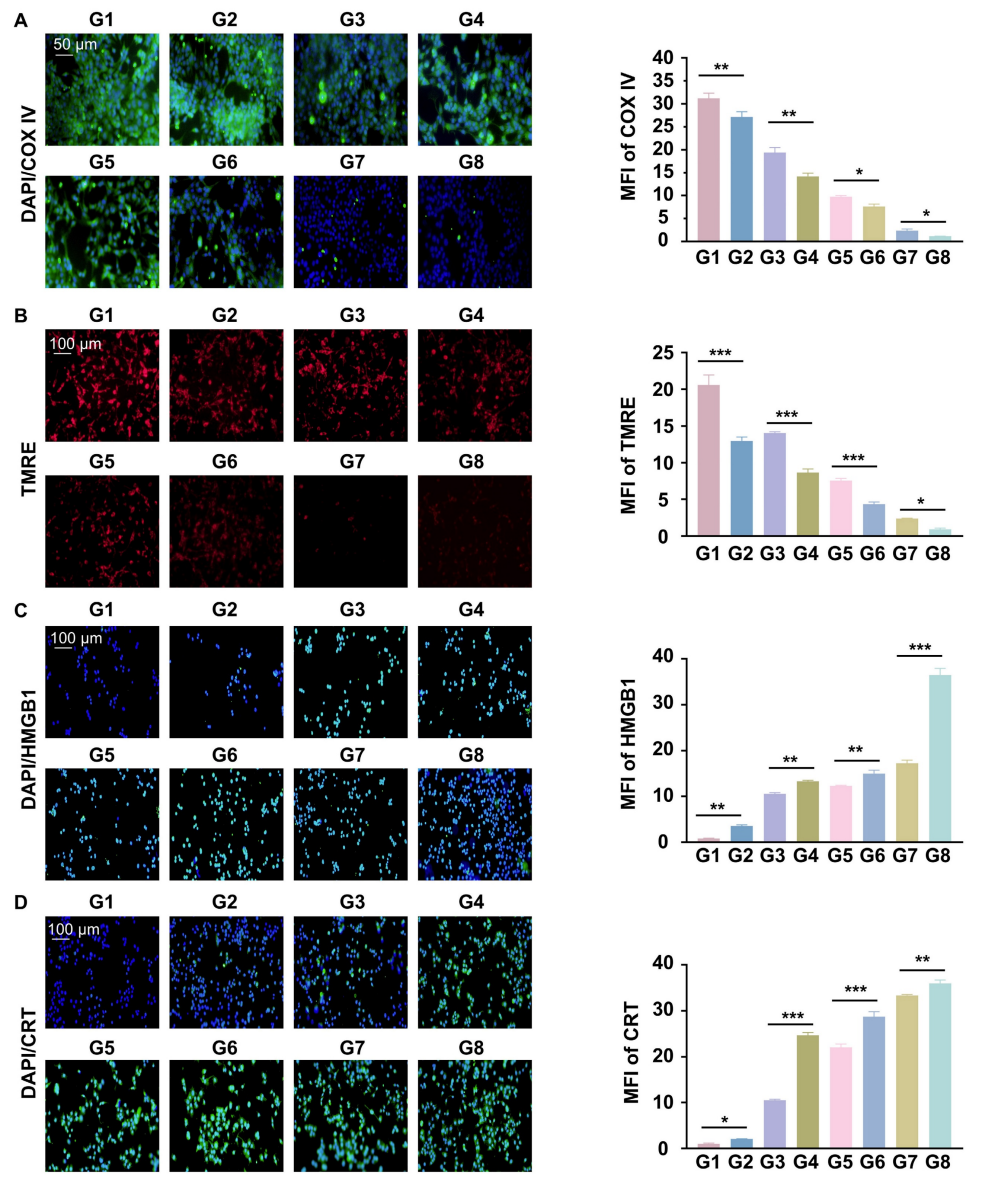
** RGD@LP-Y inhibits cellular oxygenation and induces ICD effects.** (**A**) Expression of COX IV and MFI quantitative analysis in cells treated with different materials and X-ray irradiation. (**B**) Expression of TRME and MFI quantitative analysis in cells treated with different materials and X-ray irradiation. (**C**) Expression of HMGB1 and MFI quantitative analysis in cells treated with different materials and X-ray irradiation. (**D**) Expression of CRT and MFI quantitative analysis in cells treated with different materials and X-ray irradiation. Values represent mean±SD. *P < 0.05, **P < 0.01, ***P < 0.001. ns: not significant. G1, PBS; G2, PBS+RT; G3, Y-27632; G4, Y-27632+RT; G5, LP-Y; G6, LP-Y+RT; G7, RGD@LP-Y; G8, RGD@LP-Y+RT.

Reprogrammed O2 metabolism was studied by detecting cytochrome c oxidase complex IV (COXIV), a mitochondrial chain protein. We found that RGD@LP-Y significantly reduced intracellular COX IV and that red fluorescence (TMRE), which represents the mitochondrial membrane potential, was also significantly reduced. The reduction was most pronounced after the combination of RGD@LP-Y and RT (Figure 4A, B), suggesting mitochondrial dysfunction. RGD@LP-Y may potentially reduce mitochondrial basal respiration and disrupt the mitochondrial respiratory chain, leading to decreased efficiency of the electron transport chain (ETC). When the ETC function is impaired, electron leakage increases, resulting in the formation of superoxide anions upon binding with oxygen molecules, which are subsequently converted to other ROS species 38. That may explain why RGD@LP-Y could generate a high level of ROS above.

### RGD@LP-Y induces ICD effects

A major feature of ICD is the release of CRT and HMGB1, and it can be seen that both CRT and HMGB1 levels were higher in the RGD@LP-Y group than in the other groups, and were highest in the RGD@LP-Y+RT group (Figure 4C, D), suggesting activation of a higher level of ICD.

### RGD@LP-Y activates immunogenicity by remodeling matrix stiffness

**Figure 5:**
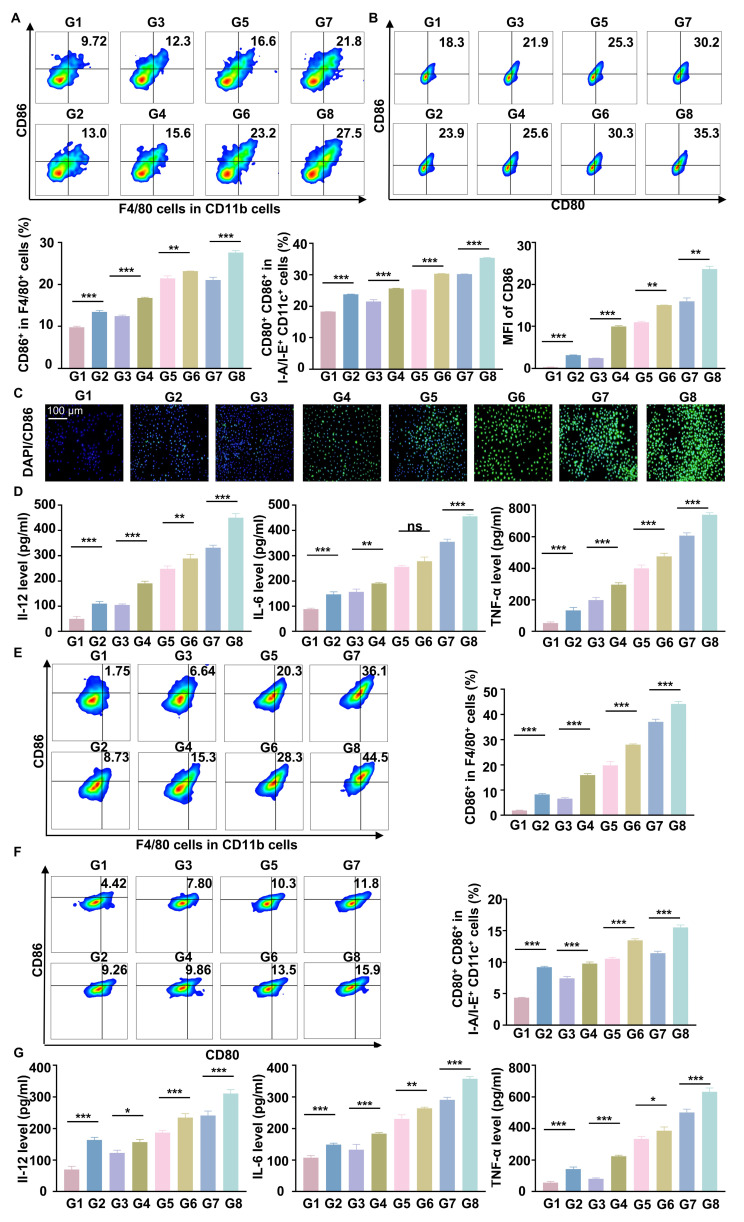
** RGD@LP-Y remodels matrix stiffness and acts immunogenicity.** (**A-B**) Representative flow cytometry plots and quantitative analysis of pro-inflammatory macrophages (CD86^+^) and mature DCs (CD80^+^/CD86^+^) in BMDMs and BMDCs treated with different materials and X-ray irradiation. (**C**) Representative IF images of CD86 and MFI quantitative analysis of pro-inflammatory BMDMs treated with different materials and X-ray. (**D**) Detection of IL-12 level in BMDC, IL-6, and TNF-α level in BMDM cell supernatant by ELISA. (**E-F**) Representative flow cytometry plots and quantitative analysis of pro-inflammatory macrophages (CD86^+^) and mature DCs (CD80^+^/CD86^+^) in THP-1 cells and BMDCs co-cultured with tumor cells preconditioned with high stiffness hydrogel, followed by treatment with different materials and X-ray. (**G**) Detection of IL-12 level in BMDC, IL-6, and TNF-α level in THP-1 cell supernatant by ELISA. Values represent mean±SD. *P < 0.05, **P < 0.01, ***P < 0.001. ns: not significant. G1, PBS; G2, PBS+RT; G3, Y-27632; G4, Y-27632+RT; G5, LP-Y; G6, LP-Y+RT; G7, RGD@LP-Y; G8, RGD@LP-Y+RT.

Subsequently, we evaluated the polarization of macrophages and the maturation of DCs using flow cytometry. We found that RGD@LP-Y+RT effectively converted macrophages into the pro-inflammatory phenotype and promoted the maturation of DCs (Figure 5A-C). Elevated levels of IL-12 secreted by DC cells, along with increased secretion of IL-6 and TNF-α (pro-inflammatory macrophage biomarkers) by macrophages, were observed (Figure 5D). This also indicates that RGD@LP-Y in combination with RT promotes DC mutation and the pro-inflammatory polarization of macrophages. Notably, these effects were also observed under high-matrix stiffness conditions (Figure 5E-G). This suggests that RGD@LP-Y+RT can activate immunogenicity and reverse the immunosuppressive effects of high matrix stiffness.

### Antitumor effects of RGD@LP-Y *in vivo*

**Figure 6:**
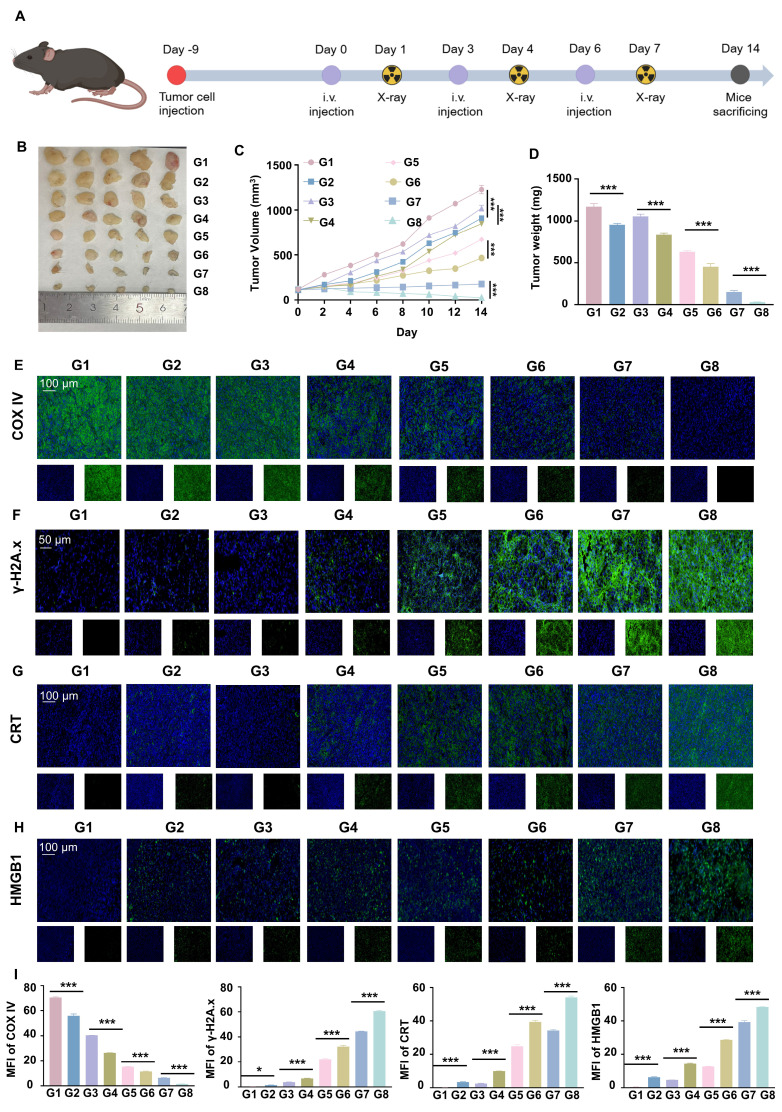
***In vivo* antitumor efficacy of RGD@LP-Y.** (**A**) Study design for RGD@LP-Y-mediated *in vivo* antitumor effects and its combination with RT. (**B-D**) Gross tumor images (**B**), tumor volume growth curves (**C**), and tumor weight (**D**) in mice after different treatments. (**E-H**) Representative IF staining images of tumor tissues for COX IV (**E**), γ-H2A.x (**F**), CRT (**G**), and HMGB1 (**H**) in mice treated with different drugs and RT. (**I**) MFI quantitative analysis of COX IV, γ-H2A.x, CRT, and HMGB1. Values represent mean±SD. *P < 0.05, **P < 0.01, ***P < 0.001. ns: not significant. G1, PBS; G2, PBS+RT; G3, Y-27632; G4, Y-27632+RT; G5, LP-Y; G6, LP-Y+RT; G7, RGD@LP-Y; G8, RGD@LP-Y+RT.

To further verify the anti-tumor effect of RGD@LP-Y *in vivo*, we established subcutaneous tumor xenografts in mice. After the tumors grew to a certain size, we administered the drug via tail vein injection on days 0, 3, and 6. Additionally, we performed RT (6Gy) on days 1, 4, and 7. Finally, on day 14, the mice were euthanized, and the tumors were removed for analysis (Figure 6A). The tumor growth curves and tumor weights indicated that RGD@LP-Y+RT significantly inhibited tumor cell proliferation compared with other groups (Figure 6B-D). Consistent with improved survival rates compared to monotherapies, the combination therapy showed enhanced outcomes. No deaths occurred in the RGD@LP-Y + RT group. The median survival time was 35 days for the RGD@LP group, 26 days for the PBS group, 31 days for the RT group, and 29 days for the Y-27632 group. RGD@LP-Y+RT significantly prolonged the survival time of tumor-bearing mice (Figure S3D).

To verify the tumor-targeting ability of RGD@LP-Y *in vivo*, we intravenously injected fluorescence-labeled RGD@LP-Y into the tail veins of mice and performed *in vivo* imaging at 1, 4, 8, 12, and 24 h post-injection. We found that RGD@LP-Y exhibited high tumor-targeting efficiency *in vivo*, with the highest accumulation at the tumor site at 12 h. By 24 h, partial clearance of RGD@LP-Y was observed (Figure S3A-C).

To further elucidate the mechanisms underlying this inhibitory effect, we conducted studies on TME and immune modulation. Compared with other groups, RGD@LP-Y+RT-treated tumor cells exhibited a marked reduction in COX IV activity (Figure 6E), which may lead to the reduction of oxygen consumption in solid tumors. Subsequent DNA damage studies revealed more intense γ-H2A.x fluorescence in the RGD@LP-Y+RT group (Figure 6F), indicating severe DNA damage. The evaluation of ICD levels showed increased exposure of CRT and release of HMGB1 (Figure 6G, H) in the RGD@LP-Y+RT group, suggesting effective ICD induction *in vivo*.

### RGD@LP-Y acts *in vivo* immunity by remodeling matrix stiffness

**Figure 7:**
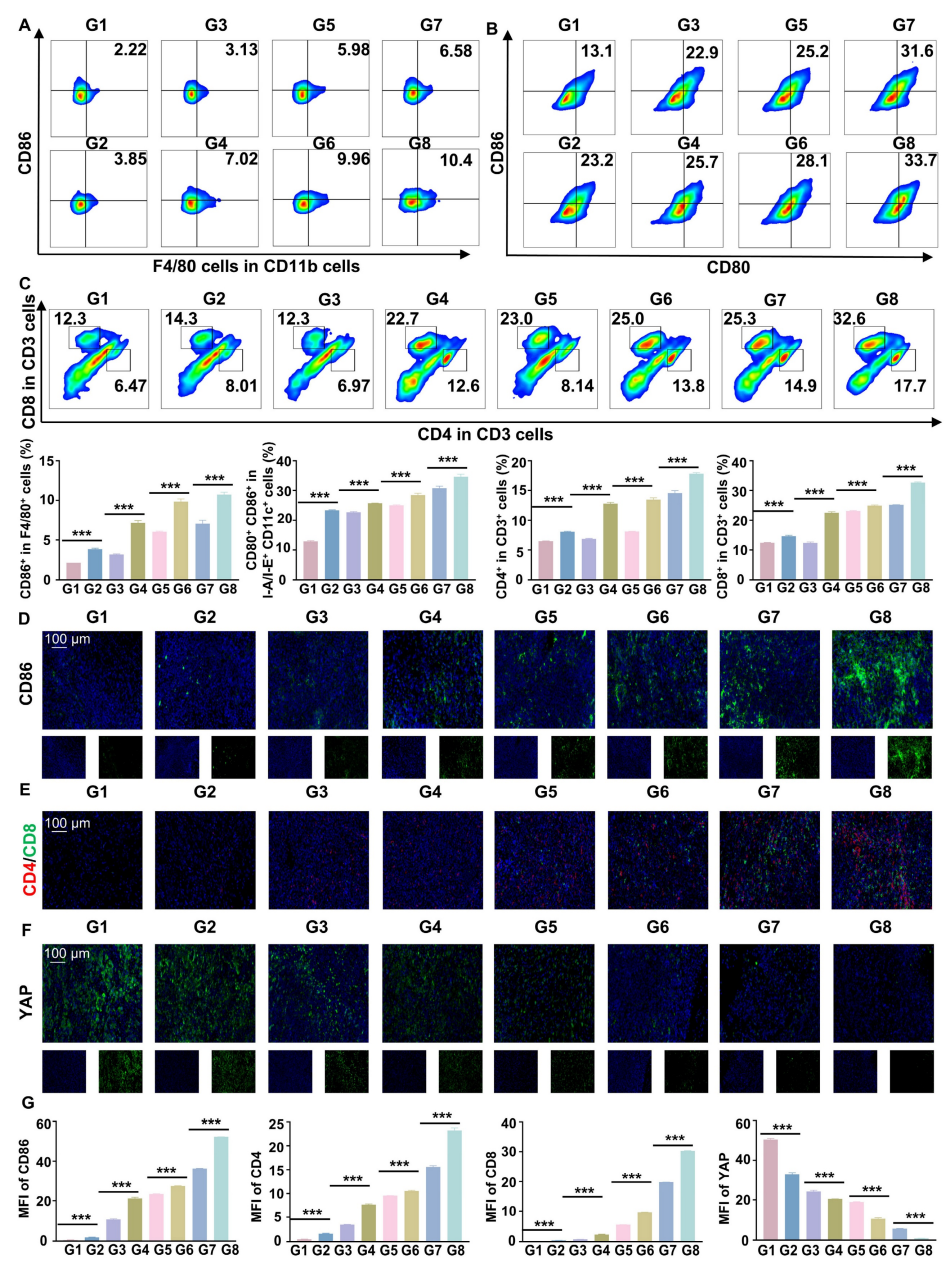
** RGD@LP-Y promotes immune activation and reduces matrix stiffness *in vivo*.** (**A-C**) Representative flow cytometry plots and quantitative analysis of pro-inflammatory macrophages (CD86^+^,** A**), mature DCs (CD80^+^/CD86^+^, **B**), and CD4^+^/CD8^+^ T cells (**C**) in mice tissues after different treatments. (**D-F**) Representative IF staining images of CD86 (**D**), CD4/CD8 (**E**), and YAP (**F**) in mice tissues following different treatments. (**G**) MFI quantitative analysis of CD86, CD4, CD8, and YAP.Values represent mean±SD. *P < 0.05, **P < 0.01, ***P < 0.001. ns: not significant. G1, PBS; G2, PBS+RT; G3, Y-27632; G4, Y-27632+RT; G5, LP-Y; G6, LP-Y+RT; G7, RGD@LP-Y; G8, RGD@LP-Y+RT.

Flow cytometry and IF staining demonstrated a significant increase in pro-inflammatory macrophages and DC maturation following RGD@LP-Y+RT treatment (Figure 7A, B, D). Additionally, effector T cells (CD4⁺ T cells and CD8⁺ T cells) were substantially elevated (Figure 7C, E), indicating robust activation of the immune response. By measuring the fluorescence intensity of YAP, a marker associated with matrix stiffness, we confirmed that RGD@LP-Y+RT significantly reduced tumor matrix stiffness (Figure 7F). Thus, RGD@LP-Y+RT may inhibit solid tumor growth and sensitize tumors to RT by activating immune responses and alleviating the immunosuppressive effects of high matrix stiffness in the TME.

### Biosafety of RGD@LP-Y

Finally, we evaluated the biosafety of RGD@LP-Y. The absence of significant effects on body weight in mice during treatment confirmed the safety of RGD@LP-Y (Figure S3F). Hemolysis assay confirmed that RGD@LP-Y exhibited good hemocompatibility, with no significant hemolysis observed (Figure S3E). In tumor-bearing mice irradiated with a dose of 6 Gy, no major organ damage was observed 15 days after treatment with RGD@LP-Y (Figure S5). Biochemical indicators also showed no significant differences compared with other groups (Figure S6). These results indicate that RGD@LP-Y exhibits favorable biosafety.

## Discussion

We successfully constructed an RGD-modified liposomal nanoplatform (RGD@LP-Y) for delivering the ROCK inhibitor Y-27632. This platform achieves tumor-specific accumulation through αvβ3 integrin targeting. It enhances HCC RT efficacy by reprogramming matrix stiffness and activating anti-tumor immunity. Experiments demonstrate that RGD@LP-Y downregulates matrix stiffness markers, activates the PI3K/AKT/NF-κB pathway, and promotes pro-inflammatory macrophage polarization and DC maturation. When combined with RT, RGD@LP-Y synergistically inhibits tumor growth and induces HCC cell apoptosis. Through suppressing mitochondrial respiration, RGD@LP-Y + RT reduces oxygen consumption, elevates ROS levels, and cause DNA damage. Then RGD@LP-Y blocks matrix stiffness-mediated signaling, and reverses immunosuppression, collectively enhancing RT-induced ICD.

The RGD sequence enables precise recognition of αvβ3 receptors, mediating nanoparticle internalization. Furthermore, RGD exhibits intrinsic immunomodulatory functions: its binding to integrins activates HMGB1-mediated ICD pathways and promotes DAMP release 34. Meanwhile, Y-27632 disrupts mitochondrial membrane potential to inhibit cellular respiration, reducing oxygen consumption and alleviating tumor hypoxia.

This process triggers ROS bursts that synergistically amplify oxidative stress with RT-generated free radicals, significantly increasing γ-H2A.X foci formation 47. Mechanistically, RGD@LP-Y interrupts matrix stiffness signaling through the integrin/Rho/ROCK axis while activating the downstream PI3K/AKT/NF-κB pathway. This dual action drives pro-inflammatory polarization of macrophages, promotes DC maturation, and enhances CD8⁺/CD4⁺ T cell infiltration. Additionally, RGD@LP-Y softens the tumor matrix to reduce physical barriers to immune infiltration, thereby reversing the immunosuppressive TME. These findings align with recent studies highlighting the critical role of reprogramming tumor-immune interactions to reignite the cancer immunity cycle and convert "cold" tumors into "hot" tumors for improved RT outcomes 48-53. Wu et al. reversed activated cancer-associated fibroblast (CAF) phenotypes and achieved matrix stiffness reprogramming to enhance cytotoxic T lymphocyte infiltration 54, further supporting the therapeutic potential of dual-pronged strategies combining matrix stiffness reprogramming with immunotherapy.

RGD@LP-Y integrates dual functionalities: targeted delivery and matrix reprogramming within a unified platform. It establishes a cascade amplification mechanism (RT sensitization to immune activation) that achieves synergistic therapy. Compared to metal-organic frameworks, the biodegradable lipid-based carrier of RGD@LP-Y eliminates organ accumulation toxicity concerns 55. Relative to conventional radiosensitizers (e.g., verteporfin), RGD@LP-Y substantially prolongs systemic circulation time. Its lipid bilayer structure enables controlled Y-27632 release, mitigating peak concentration-dependent toxicity risks 44. Besides, cyclic RGD peptides demonstrate superior stability and binding affinity, suggesting future optimization through rational ligand design (e.g., R2 peptides) could further enhance RGD@LP-Y's biocompatibility 56.

The multifunctionality of the nano platform presents new opportunities for exploring radioimmunotherapy combinations. Future research could focus on developing combinatorial strategies targeting ECM components with immune checkpoint blockade (ICB) to synergize with RT, and optimizing RGD@LP-Y synthesis for enhanced performance and safety.

## Conclusion

The RGD-modified liposomal system demonstrates superior tumor-targeting capabilities. Comprehensive biosafety evaluations confirm its excellent biocompatibility profile, positioning this nanotherapeutic approach as a promising strategy to augment RT efficacy in HCC treatment paradigms.

## Supplementary Material

Supplementary figures and tables.
