# Supplementary material for: Nanodelivery of Y-27632 by RGD-modified liposome enhances radioimmunotherapy of hepatocellular carcinoma via tumor microenvironment matrix stiffness reprogramming
Source: Theranostics. 2025 Jul 28;15(16):8569–86. doi: 10.7150/thno.114892 (PMC12374651; doi:10.7150/thno.114892)
Supplement: Supplementary file 1 — Supplementary figures and tables. [file thnov15p8569s1.pdf]

Supplementary Materials for

Nanodelivery of Y-27632 by RGD-modified liposome enhances radioimmunotherapy of hepatocellular carcinoma via tumor microenvironment matrix stiffness reprogramming

Yang Shen *et al.*

Corresponding author. Email: doctorxu120@whu.edu.cn (rm001271@whu.edu.cn), (X.X.), whuhexq@aliyun.com (H. X.).

This PDF file includes:

Table S1, S2; Figure S1-S7.

Table S1. The Sequences of the primers used in the RT-PCR.

Primer Name	Sequences
homo-IL1b-F	ATGATGGCTTATTACAGTGGCAA
homo-IL1b-R	GTCGGAGATTCGTAGCTGGA
homo-IL6-F	ACTCACCTCTTCAGAACGAATTG
homo-IL6-R	CCATCTTTGGAAGGTTCAGGTTG
homo-TNFa-F	GCCCATGTTGTAGCAAACCC
homo-TNFa-R	TGAGGTACAGGCCCTCTGAT
homo-CD80-F	AAACTCGCATCTACTGGCAAA
homo-CD80-R	GGTTCTTGTACTCGGGCCATA
homo-CD86-F	CTGCTCATCTATACACGGTTACC
homo-CD86-R	GGAAACGTCGTACAGTTCTGTG
homo-iNOS-F	TTCAGTATCACAACCTCAGCAAG
homo-iNOS-R	TGGACCTGCAAGTTAAAATCCC
homo-GAPDH-F	GGATTTGGTCGTATTGGGCG
homo-GAPDH-R	TTCCCGTTCTCAGCCTTGAC

F: forward primer, R: reverse primer, homo: Homo sapien.

Table S2. Stiffness value of polyacrylamide gel substrates.

Bis	30% Acr	2% Bis	1M HEPES	10% APS	TEMED	H2O	Total volume	Stiffness value (Young's modulus, kPa)	
%	μl	μl	μl	μl	μl	μl	μl	Primary gels	Gels added with RGD@LP-Y
0.5	1000	750	30	30	3	1187	3000	16.043±0.772	9.981±0.588

* The stiffness value was measured using atomic force microscopy (AFM).

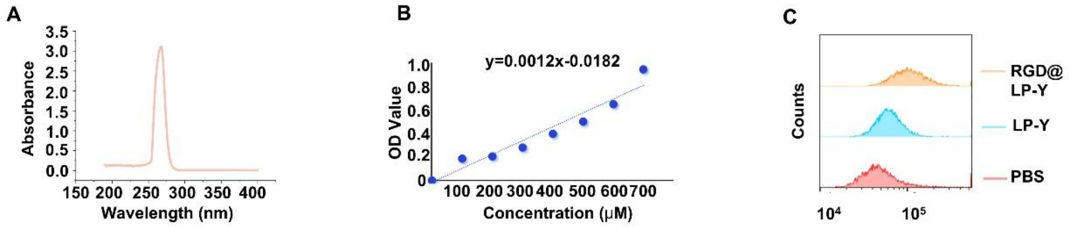

Figure S1. Characterization of Y-27632 and liposomes. (**A**) Measurement of the maximum absorption wavelength of Y-27632 drug using a UV-Vis spectrophotometer. (**B**) Standard curve of absorbance values for Y-2732 drug at 260 nm. (**C**) Representative images of absorbance of cells treated with different fluorescently labeled materials for 24 h, detected by flow cytometry.

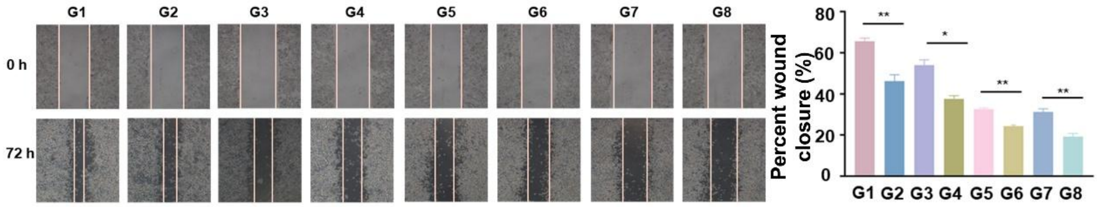

Figure S2. RGD@LP-Y and RT inhibit the migration of HCC cells. The representative images and the quantitative analysis of the wound healing assay indicated that the migration of HCC cells was inhibited after being treated with different materials and RT. Values represent mean±SD. *P < 0.05, **P < 0.01, ***P < 0.001. ns: not significant. G1, PBS; G2, PBS+RT; G3, Y-27632; G4, Y-27632+RT; G5, LP-Y; G6, LP-Y+RT; G7, RGD@LP-Y; G8, RGD@LP-Y+RT.

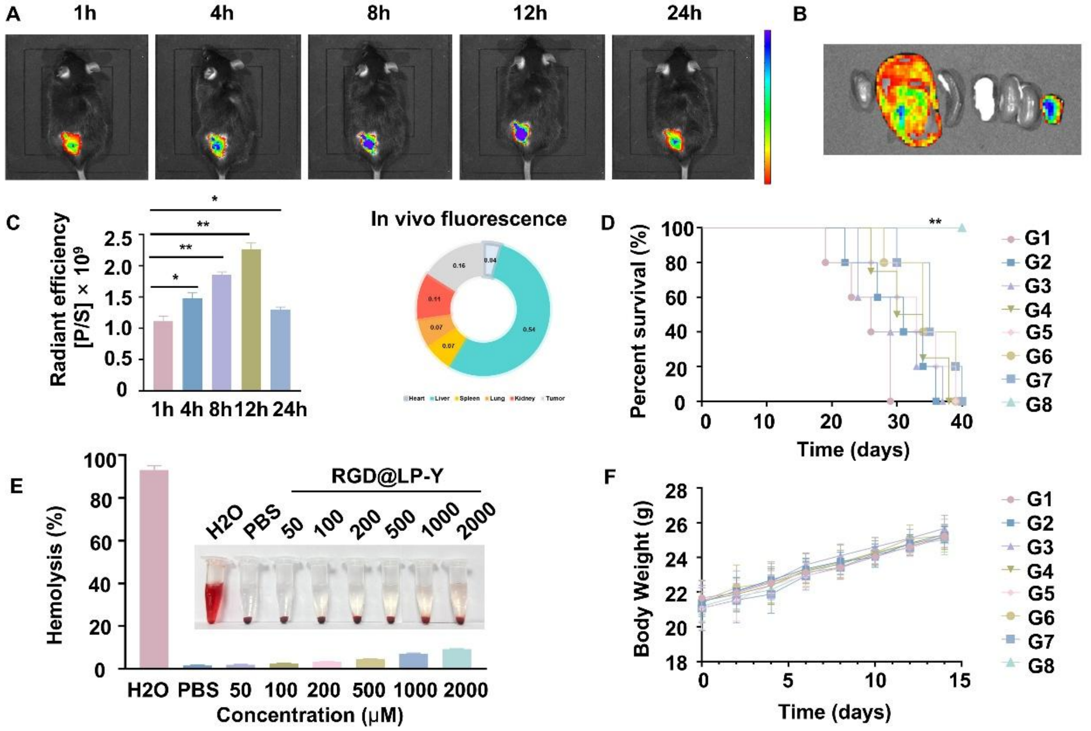

Figure S3. The tumor targeting and killing effect of RGD@LP-Y *in vivo*. (**A**) *In vivo* fluorescence imaging showing representative fluorescence intensity at tumor sites in

tumor-bearing mice following intravenous injection of 100 μg/ml fluorescence-labeled RGD@LP-Y; (**B**) Representative fluorescence intensity images of major organs and excised tumors in mice 24 h post-injection of fluorescence-labeled RGD@LP-Y; (**C**) Quantitative analysis of corresponding fluorescence intensities. (**D**) Survival curves of tumor-bearing mice with different treatments. (**E**) The hemolysis assay of RGD@LP-Y with different concentrations *in vitro*. (**F**) The body weights of mice bearing tumors after different treatments. Values represent mean±SD. *P < 0.05, **P < 0.01, ***P < 0.001. ns: not significant.

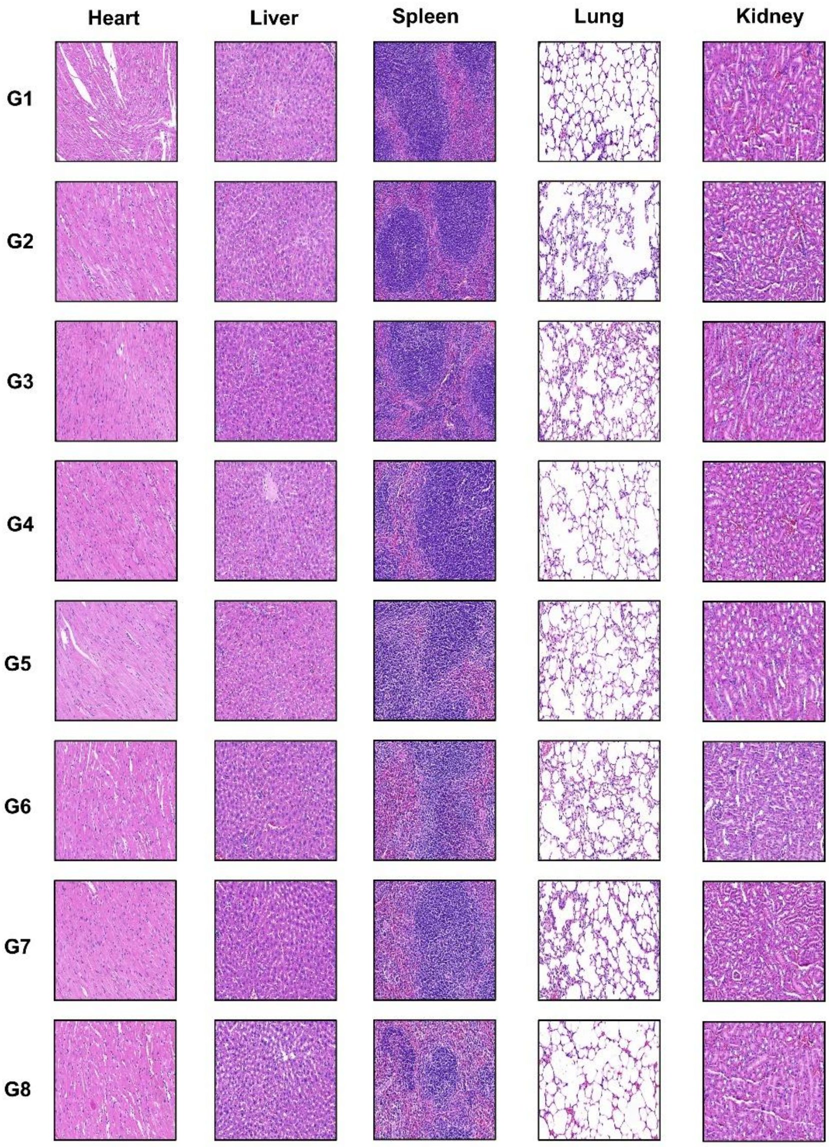

Figure S4. Biosafety analysis of RGD@LP-Y in major organs. Representative H&E staining images of major organs (heart, liver, spleen, lung, and kidney) in C57BL/6 mice after treatment with different drugs and RT demonstrated no obvious organ damage. G1, PBS; G2, PBS+RT; G3, Y-27632; G4, Y-27632+RT; G5, LP-Y; G6, LP-Y+RT; G7, RGD@LP-Y; G8, RGD@LP-Y+RT.

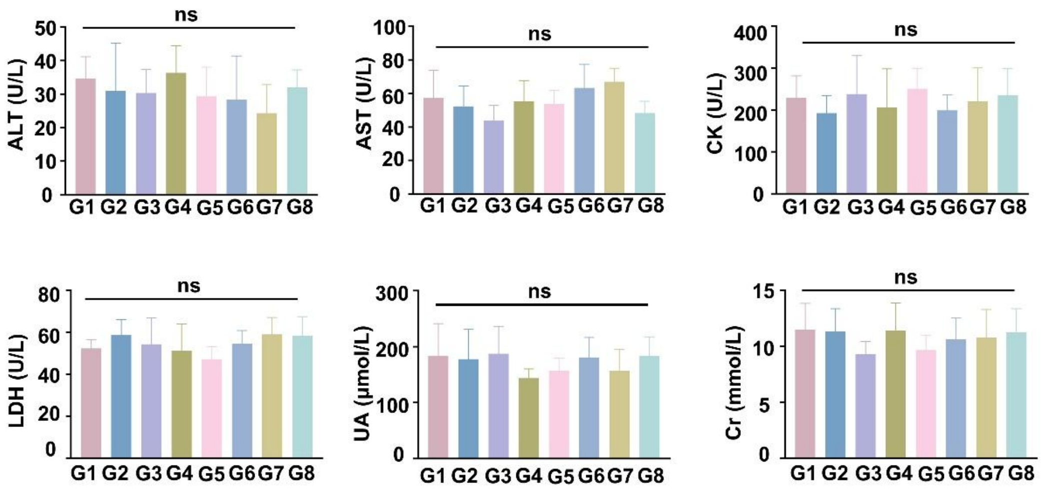

Figure S5. Biosafety analysis of RGD@LP-Y. Quantitative analysis of major blood biochemical indicators in mice treated with different drugs and RT revealed no significant differences, indicating the safety of the materials. ALT, Alanine aminotransferase; AST, Aspartate aminotransferase; CK, Creatine kinase; LDH, Lactate dehydrogenase; UA, Uric acid; CR, Creatinine; Values represent mean±SD. ns: not significant.

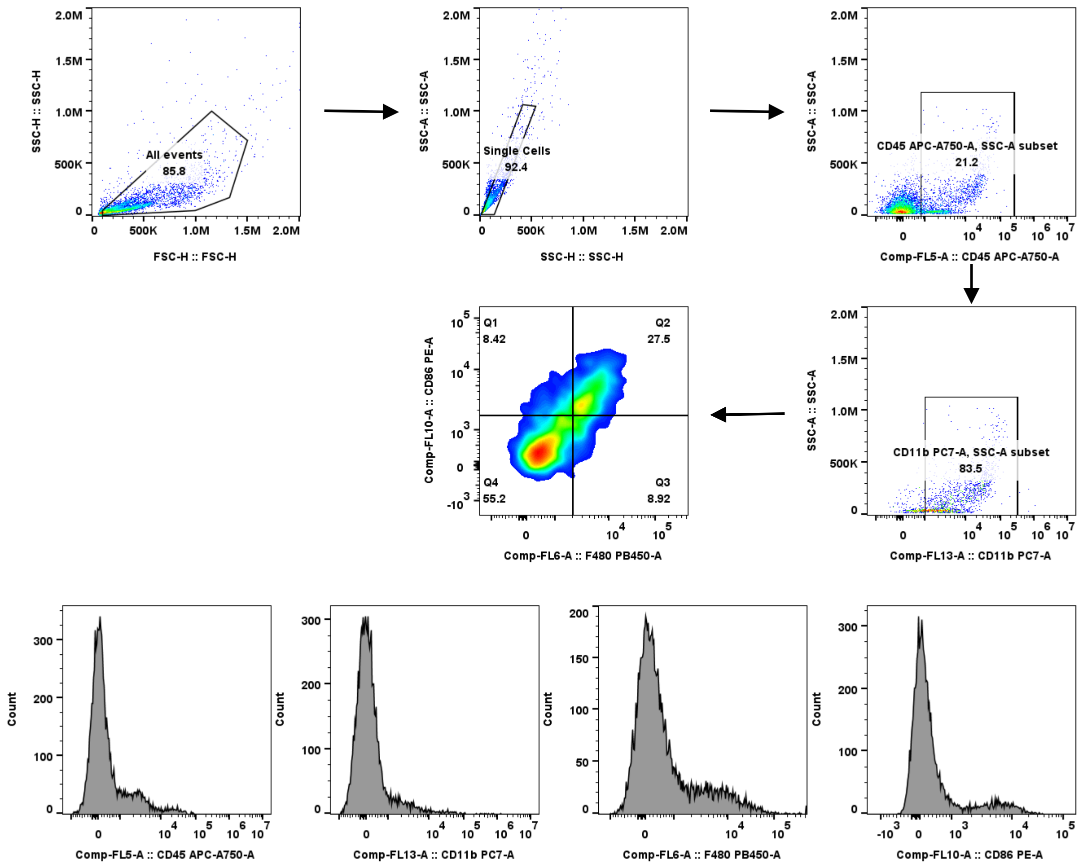

Figure S6. Flow cytometric gating strategy related to Figure 5A in the main manuscript for identifying the pro-inflammatory macrophages (CD86^{+}) in BMDMs.

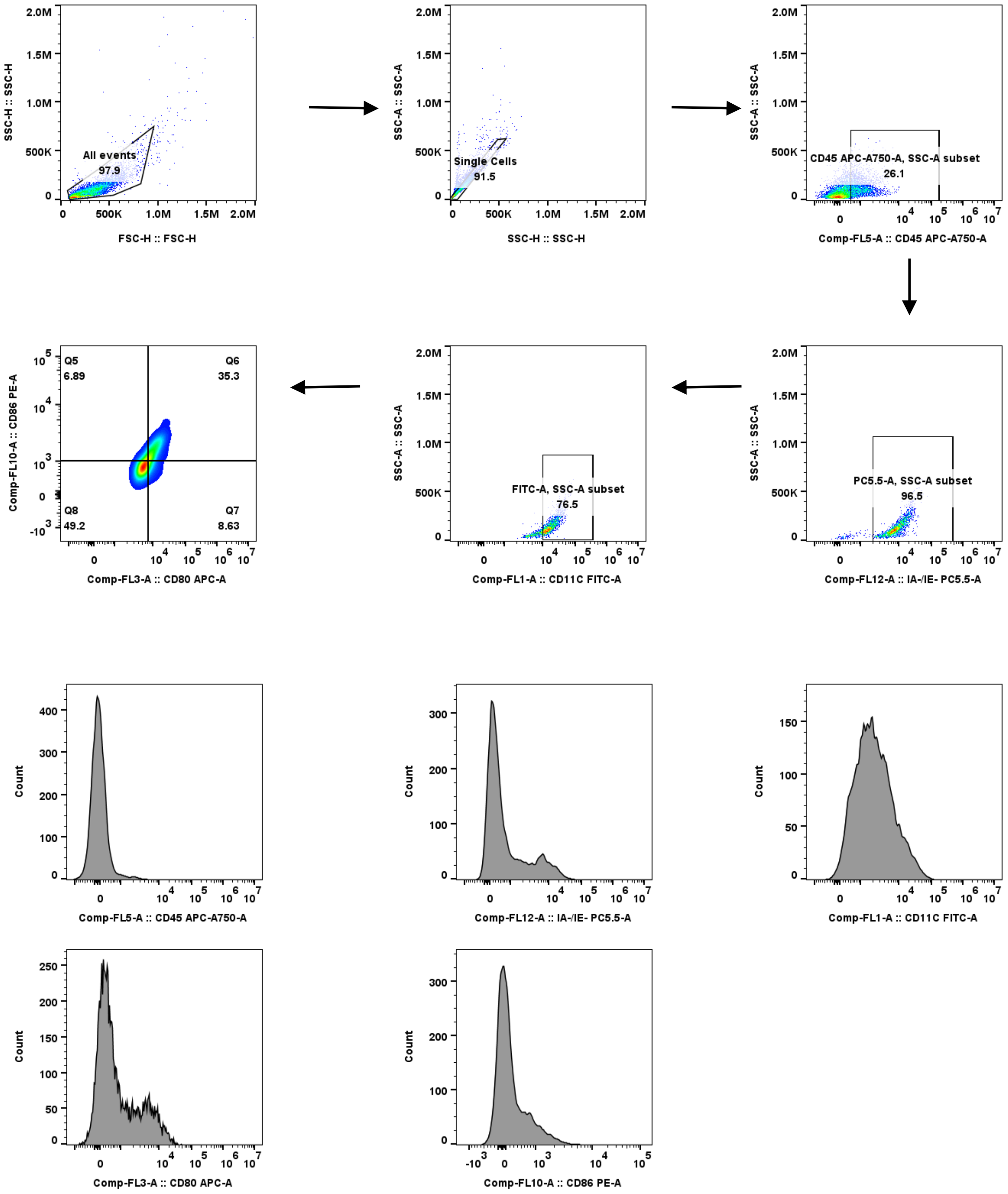

Figure S7. Flow cytometric gating strategy related to Figure 5B in the main manuscript for identifying the mature DCs (CD80^{+}/CD86^{+}) in BMDCs.
